# Seasonal variations of melatonin in ram seminal plasma are correlated to those of testosterone and antioxidant enzymes

**DOI:** 10.1186/1477-7827-8-59

**Published:** 2010-06-11

**Authors:** Adriana Casao, Igor Cebrián, Mayra Eoda Asumpção, Rosaura Pérez-Pé, José A Abecia, Fernando Forcada, José A Cebrián-Pérez, Teresa Muiño-Blanco

**Affiliations:** 1 Departamento de Bioquímica y Biología Molecular y Celular, Grupo Biología y Fisiología de la Reproduction (BIOFREZ), Instituto Universitario de Investigation en Ciencias Ambientales de Aragón (IUCA), Facultad de Veterinaria, Universidad de Zaragoza, Spain; 2 Departamento de Fisiologìa, Facultad de Ciencias de la Salud y del Deporte. Universidad de Zaragoza, Spain; 3 Departamento de Reprodução Animal de la Facultade de Medicina Veterinaria de la Universidade de Sao Paulo, Brasil; 4 Departamento de Production Animal y Ciencia de los Alimentos, Grupo Biologìa y Fisiología de la Reproducción (BIOFREZ), Instituto Universitario de Investigación en Ciencias Ambientales de Aragón (IUCA), Facultad de Veterinaria, Universidad de Zaragoza, Spain

## Abstract

**Background:**

Some breeds of sheep are highly seasonal in terms of reproductive capability, and these changes are regulated by photoperiod and melatonin secretion. These changes affect the reproductive performance of rams, impairing semen quality and modifying hormonal profiles. Also, the antioxidant defence systems seem to be modulated by melatonin secretion, and shows seasonal variations. The aim of this study was to investigate the presence of melatonin and testosterone in ram seminal plasma and their variations between the breeding and non-breeding seasons. In addition, we analyzed the possible correlations between these hormones and the antioxidant enzyme defence system activity.

**Methods:**

Seminal plasma from nine Rasa Aragonesa rams were collected for one year, and their levels of melatonin, testosterone, superoxide dismutase (SOD), glutathione reductase (GRD), glutathione peroxidase (GPX) and catalase (CAT) were measured.

**Results:**

All samples presented measurable quantities of hormones and antioxidant enzymes. Both hormones showed monthly variations, with a decrease after the winter solstice and a rise after the summer solstice that reached the maximum levels in October-November, and a marked seasonal variation (P < 0.01) with higher levels in the breeding season. The yearly pattern of GRD and catalase was close to that of melatonin, and GRD showed a significant seasonal variation (P < 0.01) with a higher activity during the breeding season. Linear regression analysis between the studied hormones and antioxidant enzymes showed a significant correlation between melatonin and testosterone, GRD, SOD and catalase.

**Conclusions:**

These results show the presence of melatonin and testosterone in ram seminal plasma, and that both hormones have seasonal variations, and support the idea that seasonal variations of fertility in the ram involve interplay between melatonin and the antioxidant defence system.

## Background

Melatonin plays a central role in fine-tuning circadian rhythms [1] and seasonal changes [2] through its daily nocturnal increase in the blood [3]. In seasonally breeding mammals that use changes in the photoperiod to time their reproductive cycles, temporal signals to the reproductive system are controlled by the daily rhythm in melatonin production [4-7]. Certain breeds of sheep are highly seasonal in terms of reproductive capability, regulated by photoperiod and melatonin secretion [8,9]. Therefore, seasonal melatonin variation has been thoroughly studied in this specie, and although seasonality is less marked in male than in female, changes in testicular volume, hormonal profiles, sexual behaviour and semen quality that affect the reproductive performance of rams have been reported [10-14].

Rasa Aragonesa is a local Spanish genotype with a short seasonal anoestrous period (< 100 days) between May and July [15]. In previous studies on this breed, we showed that the treatment of rams with slow release implants of melatonin during the non-breeding season accounted for increased scrotal diameter and improved the reproductive performance of ewes naturally mated [16] or inseminated during anoestrus with semen from these melatonin-implanted males [17]. Likewise, we have recently demonstrated a beneficial direct action of melatonin on sperm motility [17] and on other ram sperm characteristics during the non-breeding season, with decreased apoptotic-like changes and modulating capacitation [18].

Oxidative stress is defined as an imbalance between the cellular antioxidant defense systems and the production of reactive oxygen species (ROS) [19]. The antioxidant ability to scavenge ROS of mammalian sperm and seminal plasma allows maintaining the balance between ROS generation and neutralization. We have shown certain seasonal changes in the activity of the antioxidant enzyme defence system in ram seminal plasma [20] which could partly explain the seasonal variations in fertility observed in the ram [21]. In addition, the activity and expression of antioxidant enzymes seem to be modulated not only by the oxidant status of the cell but also by other factors like the presence of melatonin [22,23]. Therefore, we could hypothesize that seasonal difference in ram sperm quality might be regulated by the presence of melatonin in seminal plasma along with antioxidant enzyme activity which could prevent the oxidative damage of spermatozoa.

To further the understanding of the melatonin influence in ram semen, in this study we determined the presence of melatonin and testosterone in ram seminal plasma and investigated their variations between the breeding and non-breeding seasons. In addition, we analyzed the possible correlations between these hormones and the antioxidant enzyme defence system, comprising superoxide dismutase (SOD), glutathione reductase (GRD), glutathione peroxidase (GPX) and catalase (CAT).

## Methods

### Experimental design and semen collection

We determined the level of melatonin and testosterone, and the activity of the antioxidant enzyme defence system in ram seminal plasma throughout the year by weekly analysis. Correlations between the levels of both hormones and the activity of superoxide dismutase (SOD), glutathione reductase (GRD), glutathione peroxidase (GPX) and catalase (CAT) were also determined, as well as variations between breeding and non-breeding seasons.

Semen was collected from nine Rasa Aragonesa rams maintained under uniform nutritional conditions at the Experimental Farm of the University of Zaragoza, Spain (latitude 41° 41' N, under Mediterranean climate conditions), in compliance with the requirements of the European Union for Scientific Procedure Establishments. All experimental procedures were performed under the supervision of the Ethics Committee of the University of Zaragoza. All the rams belonged to the National Association of Rasa Aragonesa Breeding (ANGRA) and were 2-4 years old. The sires were kept apart, and semen was collected every two days, in two successive matings each day. First and second ejaculates were pooled separately to obtain a uniform, good quality sperm sample suitable for representative studies of ram semen, according to previous report [24]. Only first ejaculate samples were used in this study.

### Seminal plasma extraction

Seminal plasma was extracted from pooled first ejaculates by centrifugation at 2.400 × g for 10 min in a microfuge at 4°C. The supernatant was centrifuged again at 2.400 × g for 10 min, and seminal plasma was recovered and, after filtering through a 0.22 μm Millipore membrane (Millipede Ibérica, Madrid, Spain) was kept at -20°C until analysed. Two seminal plasma samples obtained per week were pooled and analyzed.

### Melatonin evaluation

Melatonin values in ram seminal plasma were measured by means of a commercial competitive immunoassay (Direct saliva melatonin ELISA kit, Bühlmann Laboratories AG, Switzerland, sensitivity: 0.5 pg/ml, intro-assay variability: 5.2%), following the manufacturer's instructions. Briefly, 100 μl of each sample, control and calibrator were loaded in duplicate in a microtiter plate coated with an anti-melatonin antibody, and incubated for 16-20 h at 2-8°C. After incubation, 50 μl of biotinylated melatonin were added to each well and incubated for 3 h at 2-8°C. After three washes, 100 μl of streptavidin conjugated to horseradish peroxidase (HRP) were loaded to the wells and incubated for a further 60 min in a plate rotator set at 600 rpm at 18-28°C. Wells were washed three times again, and 100 μL of tetramethylbenzidine substrate (TMB) were added to each well and incubated for 30 min on a plate rotator at 600 rpm and 18-28°C and protected from direct light. After incubation, 100 μl of 0.25 M SO_4_H_2 _solution were added and absorbable was measured on a microtiter plate reader (TECAN Spectrafluor plus, Switzerland) at 450 nm.

### Testosterone assays

Testosterone evaluation in ram seminal plasma was performed by means of a total testosterone commercial ELISA kit assay (Testo-Easia, BioSource Europe, S.A., Belgium. sensitivity: 0.05 ng/ml, intro-assay variability: 4.8%), following the manufacturer's instructions. Briefly, 50 μl of each sample, control and calibrator, along with 100 μl of testosterone labeled with horseradish peroxidase (HRP) were loaded in duplicate in a microtiter plate coated with an anti-testosterone specific antibody, and incubated for 1 h at room temperature. After incubation, wells were washed three times, and 100 μl of chromogenic substrate (TMB) were added to each well and incubated for 30 min at room temperature, protected from direct light. After incubation, 100 μl of 0.2 M HCl solution were added and absorbance was measured on a microtiter plate reader (TECAN Spectrafluor plus, Switzerland) at 450 nm.

### Antioxidant enzymes assays

Antioxidant enzymatic activity of four enzymes: superoxide dismutase (SOD), glutathione peroxidase (GPX), gluthatione reductase (GRD) and catalase was determined.

#### Superoxide dismutase (SOD)

SOD activity was measured by optimization of the method that we used previously [20]. Enzymatic activity was measured as a decrease of the XTT (3'-(1-[(Phenylamino)-carbonyl]-3,4-tetrazolium)-bis(4-methoxy-6-nitro) benzenesulphonic acid hydrate) reduction by superoxide anion generated by xanthine oxidase.

The SOD activity was assessed as the competition between reaction c and b which is measured as a decrease of the rate of XTT reaction. The reaction mixture contained 40.5 mM sodium phosphate buffer pH 7.8; 0.15 mM xanthine; 0.15 mUI xanthine oxidase, 30 mM XTT and 20 μl of sample to complete a final volume of 1 ml. The reaction was initiated by the addition of xantine oxidase, and the absorbable change at 470 mm was monitored for 3 min with a Hitachi spectrophotometer (U-2000). One enzyme unit (IU) is defined as the amount of SOD capable of transforming 1.0 mmole/min of O_2_^•¬^

#### Glutathione peroxidase (GPX)

GPX activity was measured by using a variation of the method previously used [20] based on that described by Paglia and Valentine [25]. Enzymatic activity was measured following the oxidation of glutathione (GSH) to oxidized glutathione (GSSG) catalysed by GPX and using as an electron acceptor ter-Butylhydroperoxide (t-BuO_2_H), coupled to the recycling of GSSG back to GSH utilizing GRD and NADPH.

The reaction mixture contained 300 mM sodium phosphate buffer pH 7.2; EDTA 0.5 mM, 54 mUI of GRD; 85 μM NADPH; 2 mM GSH; 1.2 mM t-BuO_2_H and 30 μl seminal plasma to complete a final volume of 1 ml. The absorbance change at 340 nm was monitored for 3 min with a Hitachi spectrophotometer (U-2000). One unit will cause the oxidation of 1.0 mole/min of NADPH.

#### Glutathione reductase (GRD)

GRD activity was measured,by using a variation of the method described by Goldberg and Spooner [26]. Enzymatic activity was measured following the decrease in absorbance at 340 nm due to NADPH oxidation as a consequence of the GSSG reduction. The reaction mixture contained 300 mM sodium phosphate buffer pH 7.2; 0.5 mM EDTA; 85 μM NADPH; 0.8 mM oxidized glutathione (GSSG) and 50 μl seminal plasma to complete a final volume of 1 ml. The absorbable change at 340 nm was monitored for 3 min with a Hitachi spectrophotometer (U-2000). One unit will cause the oxidation of 1.0 mmole/min of NADPH.

#### Catalase

Catalase activity was measured by using a variation of the method described by Marti et al. [20]. The enzymatic activity was determined by the decrease in absorbance due to H_2_O_2 _reduction to H_2_O and O_2 _in catalase presence.

The reaction mixture contained 50 mM sodium phosphate buffer (pH 7); 30 mM H_2_O_2 _and 30 μl seminal plasma to complete a final volume of 1 ml. The absorbance change at 240 nm was monitored for 30 sec with a Hitachi spectrophotometer (U-2000). One enzyme unit (IU) is defined as the amount of catalase capable of transforming 1.0 μmol/min of H_2_O_2_.

### Statistical analysis

Monthly and seasonal results are shown as mean ± S.E.M. of the number of samples assessed in each case. Distribution of the data was evaluated by the Kolmogorov-Smirnov test. Because melatonin data were not normally distributed, and had a log normal distribution, logarithm transformation of these data was carried out to perform statistical analysis.

Differences between breeding (August-February) and non-breeding (March-July) seasons were compared by means of an analysis of variance (ANOVA) test, and correlations between assessed parameters were compared by means of Pearson's bivariated correlation test. When correlation between parameters was significant, linear regression test were carried out. All statistical analysis was performed using SPSS (v.15.0) software.

## Results

All studied samples presented measurable quantities of melatonin, testosterone and antioxidant enzymes.

**Figure 1:**
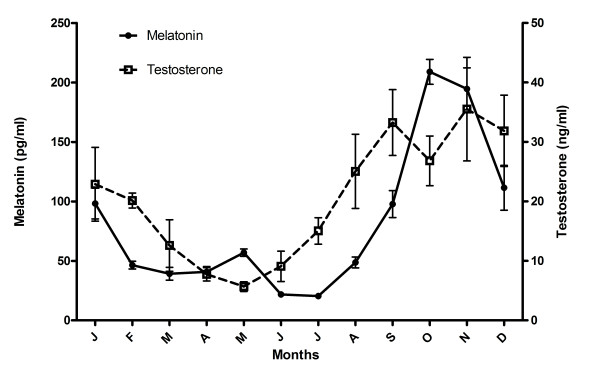
**Monthly values of melatonin and testosterone in ram seminal plasma**. Values are shown as mean ± S.E.M. of 4 seminal plasma samples/month.

Both hormones showed monthly variations, with a decrease after the winter solstice that reached the minimum in May (testosterone) or June and July (melatonin), and a rise in August that reached the maximum levels in October-November (Fig. 1, P < 0.01). The variation of melatonin levels in ram seminal plasma was higher than that of testosterone, with low values (less than 50 pg/ml) from March to July while from September to January it doubled or tripled this value (> 100 pg/ml, P < 0.01). A peak of a mean value of 208.95 ± 10.45 pg/ml was found in October. Testosterone content in ram seminal plasma reached the minimum in May, increasing thereafter, and achieved the maximum value of 35.52 ± 8.71 ng/ml in November (Fig. 1, P < 0.01). Also, melatonin and testosterone values showed a marked seasonal variation in ram seminal plasma (P < 0.01, Table 1), with low levels in the non-breeding season and high concentrations in the breeding one.

**Table 1:** Seasonal values of melatonin, testosterone and antioxidant enzyme activities in ram seminal plasma

	Breding season	Non breeding season
	(n = 28)	(n = 20)
Melatonin (pg/ml)	137.51 ± 17.8^a^	46.57 ± 8.37^b^
Testosterone (ng/ml)	28.13 ± 3.35^a^	10.66 ± 2.92^b^
GPX (nmole/min.ml)	61.57 ± 4.48	52.86 ± 8.53
GRD (nmole/min.ml)	11.82 ± 0.84^a^	7.5 ± 0.47^b^
SOD (mmole/min.ml)	8.86 ± 0.15	8.56 ± 0.26
Catalase (μmole/min.ml)	2.11 ± 0.25	1.74 ± 0.19

Values are shown as mean ± S.E.M. of the number of the number of seminal plasma samples assessed in brackets. Different letters in the same row account for significant differences between seasons (*P *< 0.01)

**Figure 2:**
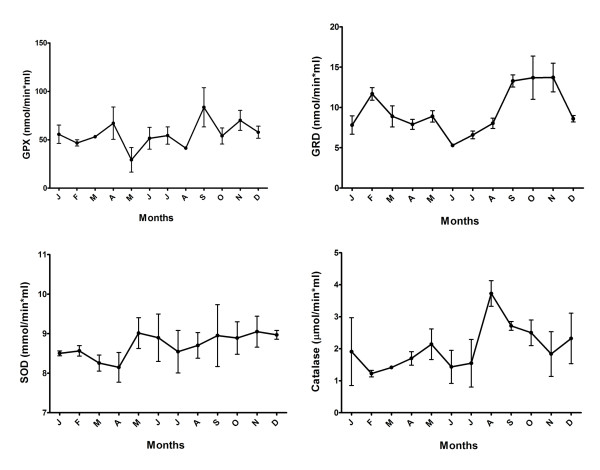
**Monthly distribution of GRD, GPX, CAT, and SOD activities in ram seminal plasma**. Values are shown as mean ± SEM of 4 seminal plasma samples/month.

The yearly pattern of the four antioxidant studied enzymes is shown in Fig. 2. GRD activity was minimum in June and maximum in October- November (P < 0.01), very close to the melatonin pattern. Likewise, the distribution of catalase was also close to that of melatonin, although the maximum value was achieved in August (P < 0.05) decreasing thereafter. GPX and SOD activities were spread out along the year (Fig. 2).

Among the four antioxidant enzymes assessed, only GRD showed a significant seasonal variation (P < 0.01) with a higher activity during the breeding season (Table 1). GPL and SOD activities did not show a seasonal distribution, and although marked monthly variations were found in catalase distribution, it was not significant (P = 0.11)

**Figure 3:**
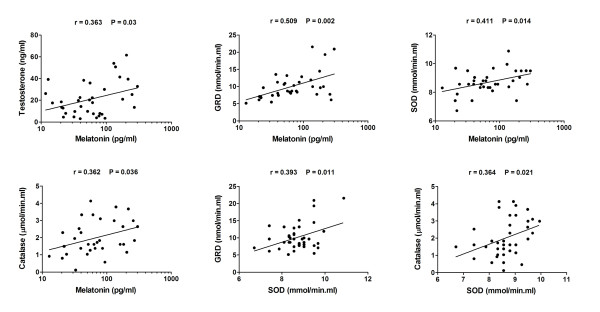
**Graphical representation (scattered plot) of correlation and linear regression fit between hormones and antioxidant enzymes in 48 seminal plasma samples**.

Correlation analysis between the studied hormones and antioxidant enzymes showed a significant correlation between melatonin and testosterone, GRD, SOD and catalase. In addition, SOD showed a direct correlation with GRD and catalase, but not with testosterone or GPX (Table 2). A graphical representation of correlations and linear regression fit is sown in Fig. 3.

**Table 2:** Pearson's correlations (r) between melatonin, testosterone and antioxidant enzymes in ram seminal plasma

	Melatonin	Testosterone	GPX	GRD	SOD	CAT
Melatonin	1	0.363*	-0.009	0.509**	0.411*	0.362*
Testosterone	0.363*	1	0.175	0.318	0.209	0.223
GPX	-0.009	0.175	1	0.243	-0.175	-0.264
GRD	0.509**	0.318	0.2431	1	0.393*	0.176
SOD	0.411*	0.209	-0.175	0.393*	1	0.364*
CAT	0.362*	0.223	-0.264	0.176	0.364*	1

*P < 0.05

**P < 0.01

## Discussion

The results of this study show the presence of melatonin and testosterone in ram seminal plasma, both hormones with a seasonal variation, in the same way as occurs in blood serum throughout the year [27,28]. In order to investigate the hypothesis that melatonin is functionally involved in the antioxidant defence system, we determined the activity of the main antioxidant enzymes in the same sample. The obtained data demonstrate a strong correlation between melatonin and testosterone, as well as between melatonin and the activity of three antioxidant enzymes (GRD, SOD and catalase) in ram seminal plasma, which supports the melatonin role in the regulation of both the male reproductive [29,30] and the antioxidant enzyme defense [23] systems.

Melatonin is able to modulate the reproductive physiology in photoperiod-dependent seasonally breeding mammals [4-7]. Melatonin variation in ram seminal plasma shown here seems to reflect the seasonal variation of melatonin secretion by the pineal gland [31]. Given that the breeding season in sheep is regulated by photo period and melatonin [32], both seasonal [11,12] or melatonin [14,30,33] effects on reproductive parameters in rams have been largely studied. Detrimental effects of long days, and beneficial effects of both short days or melatonin treatment in the non-breeding season were supposed to be due to the melatonin regulator effect on the hypothalamus-pituitary-testicular axis [27], modulating GnRH pulsativility [13] and gonadotropin and testosterone production [34,35]. However, we have recently proved a direct action of melatonin on ram spermatozoa, decreasing sperm apoptotic-like features and modulating sperm capacitation and fertilization rates [18]. Therefore, the high variation in melatonin concentration throughout the year in ram seminal plasma found in this study could partly explain differences in sperm quality and fertility observed between the breeding and non-breeding seasons [10,16,17].

Similarly, testosterone levels also showed seasonal variation in ram seminal plasma, and they were significantly higher during the breeding season, which could influence fertility. Testosterone, produced by testes, is required for maturation of male germ cells and sperm production and quality [36]. Testosterone is metabolized to estrogens by aromatase [37], and estrogens seem to regulate ejaculated sperm motility [38]. The presence of testosterone and estrogen receptors in human spermatozoa [39,40] and the strong correlation found between the aromatase expression and motility in human [41] and buffalo [42] ejaculated sperm suggest that aromatase could be involved in the modulation of sperm motility by metabolization of seminal plasma testosterone into estrogens. Therefore, the higher ram sperm quality [43] and fertilization rate [16] observed during the breeding season could be partially caused by high levels of testosterone in ram seminal plasma, which could be transformed into estrogens by aromatase and improve motility parameters. This theory is supported by our previous results which showed that melatonin implants in ram during the non-breeding season increased sperm progressive motility [17], although direct in vitro incubation of ram spermatozoa with different melatonin doses during the non-breeding season did not affect sperm motility [18]. The possibility that variations in motility parameters observed in ejaculates of melanin-implanted rams are due to an increase in locally produced estrogens by aromatase, as a result of high testosterone levels in seminal plasma, cannot be ruled out.

Seasonal variation of testosterone in ram seminal plasma is slightly lower than that of melatonin. Given that breeds can differ in their fluctuations of gonadotropin and testosterone levels in response to changes in day length and melatonin secretion [27], the minor testosterone variation may be a reflection of the short non-breeding season of Rasa Aragonesa breed [15]. The relationship between melatonin and testosterone has been well documented. The effect of melatonin on blood testosterone levels has been reported in various species, including ovine. Testosterone blood levels in ram are known to fluctuate throughout the year [27,34], and are increased by melatonin treatment during the non-breeding season [33]. The high correlation found in this study between the levels of melatonin and testosterone in ram seminal plasma is worth pointing out, and suggests that both hormones are also closely related in this fluid, and could reflect changes in these blood hormones throughout the year.

On the other hand, spermatozoa are very sensitive to oxidative stress effects, and their fertilizing capacity is impaired due to early apoptosis and DNA damage [44]. To protect spermatozoa from ROS, epydidimal epithelium [45] and accessory sex glands secrete antioxidant enzymes as well as other free radical scavengers [46]. GRD, GPX and SOD are mainly secreted by the prostate, while catalase would be of multi-glandular origin [47]. Antioxidant enzyme activity shows endogenous daily cycles that may be regulated by Circadian melatonin rhythms [48].

In this study, we have found a seasonal variation of GRD in ram seminal plasma with significantly higher activity in the breeding season. The seasonal variation of GRD in ram seminal plasma found in this study, and the strong correlation between melatonin and GRD SOD and catalase suggest a seasonal regulation of these antioxidant enzymes by melatonin. The detection of melatonin receptor in rat epididymis [49] suggests a role for melatonin in the regulation of epididymis antioxidant enzyme production [23]. However, there is still no evidence of melatonin regulation of prostatic antioxidant enzyme production. Although melatonin receptors have been evidenced in human and rat prostate benign tumours [50,51], there are no reports of melatonin receptors in prostate of healthy males, which might mediate the prostatic production of antioxidant enzymes. However, the lipophilic nature of melatonin would allow this hormone to cross the plasma membranes, and thus its possible stimulatory effect on antioxidant enzyme production could be mediated by nuclear or cytosol binding sites [52]. Furthermore, melatonin is supposed to regulate antioxidant enzyme activity via melatonin plasma membrane receptors MT_1_/MT_2 _[23], increasing messenger RNA and protein levels of these enzymes [22]. Additionally, along with the direct effect of the antioxidant enzyme defence system, the high ability of melatonin to function in the reduction of oxidative stress could also involve the prevention of toxic effects of ROS in seminal plasma [53], as this indolamine directly neutralizes a high number of toxic free radicals [54].

## Conclusions

In conclusion, this study demonstrates the presence of melatonin and testosterone in ram seminal plasma, and that both hormones have seasonal variations. The obtained results support the idea that melatonin is involved in the regulation of semen quality and the antioxidant enzyme activity that affect the reproductive performance of rams, and that seasonal variations of fertility in the ram involve an interplay between melatonin and the antioxidant defence system. Further investigations on this subject would be applicable to animal reproductive biology.

## Competing interests

The authors declare that they have no competing interests.

## Authors' contributions

JACP and AC designed the research, AC performed melatonin and testosterone assays and drafted the manuscript, IC and MEODAA performed antioxidant enzymes assays, RPP and TMB carried out data analysis and interpretation, and TMB, FF and JAA revised and approved the article. All authors read and approved the final manuscript.
